# “But My Speech Is Fine...”: Expanding the Speech-Language Pathologist’s Role in Nursing Home Dementia Care

**DOI:** 10.1093/geroni/igaf122.173

**Published:** 2025-12-31

**Authors:** Natalie Douglas

**Affiliations:** University of Louisiana at Lafayette, Lafayette, Louisiana, United States

## Abstract

Speech-language pathologists are uniquely positioned to support communication and person-centered care for people living with dementia in nursing homes. A recent feasibility study demonstrated that Dementia Collaborative Coaching can be implemented by speech-language pathologists to train certified nursing assistants in communication strategies within usual care workflows. In that study, four speech-language pathologists implemented coaching with 10 nursing assistants and 15 residents across 90 sessions, achieving 64% fidelity and significant reductions in behavioral distress, as measured by the Cohen-Mansfield Agitation Inventory, t(14) = 10.51, p < .001, d = 2.76. This presentation builds upon those findings by adapting Dementia Collaborative Coaching for a nursing home in southwestern Louisiana, located in one of the most socioeconomically disadvantaged regions in the United States, ranking in the 9th decile on the Area Deprivation Index. Adaptations included reducing session frequency to improve feasibility, training multiple nursing assistants per resident to ensure continuity across shifts, and incorporating culturally responsive materials tailored to the linguistic and social diversity of the region. Speech-language pathologists developed relationships with nursing assistants, modeled skilled communication strategies, and fostered a collaborative learning environment to integrate communication-focused care into daily routines. Preliminary findings suggest these adaptations improved nursing assistant engagement, consistency in strategy use, and resident interaction quality. This study highlights how speech-language pathologists can modify evidence-based interventions to fit resource-limited nursing homes while maintaining fidelity and impact.

